# Reducing Repeat Prescriptions (FP10s) Issued by South Hub Community Mental Health Team: A Quality Improvement Pilot

**DOI:** 10.1192/bjo.2026.11500

**Published:** 2026-06-30

**Authors:** Aashiana Thiyam, Nurul Yahya

**Affiliations:** 1 Birmingham and Solihull Mental Health Trust, Birmingham, United Kingdom; 2 Priory Hospital, Birmingham, United Kingdom

## Abstract

**Aims:**

Repeat FP10 prescribing from secondary care increases administrative burden, risks medication gaps for patients, and may delay timely access to treatment. The South Hub (Yewcroft) team initiated a QI project to streamline repeat prescribing processes and increase timely initiation of Enhanced Shared Care Agreements (ESCAs) so that stable patients could be managed by primary care.

By 31 January 2025, we aimed to reduce the weekly frequency of repeat FP10 issues for patients under Yewcroft CMHT by 20%.

**Methods:**

A multidisciplinary project team used Plan–Do–Study–Act(PDSA) cycles to test multiple change ideas across four domains: (1) facilitate use of the electronic prescribing and medicines administration system (EPMA) for FP10 generation; (2) improve accuracy and accessibility of repeat-prescription tracking; (3) strengthen liaison with GPs through timely ESCA initiation and repatriation letters; and (4) introduce a duty doctor rota to improve prescribing responsiveness. Process measures included weekly counts of repeat FP10s, reception calls about FP10s, and weekly antipsychotic FP10s. Baseline data were collected over eight weeks (median 6 repeat FP10s/week).

**Results:**

Multiple interventions were implemented, including EPMA prompts, printer-use guidance, an edited shared spreadsheet for tracking, repatriation letter templates, and a duty rota. Data collection revealed intermittent peaks and troughs without a sustained shift in the primary outcome during the project period. Operational challenges limited impact: variable staff access to systems, frequent staff rotation, printer failures, and constrained administrative capacity impeded consistent implementation and reliable data capture. Despite not meeting the 20% reduction target, the project improved local prescribing processes, increased awareness of ESCA pathways, and produced practical tools (templates, tracking spreadsheet) that the team judged beneficial.

**Conclusion:**

Rapid introduction of multiple changes in a resource-limited context made it difficult to demonstrate measurable improvement within the project timeframe. Future work should adopt a phased approach, secure protected data-collection capacity, prioritise system access for prescribers, and include ESCA-signed rates as an additional outcome. With these adjustments, the interventions piloted here have potential to reduce repeat FP10 burden and improve continuity of care.

